# A Review of Translational Animal Models for Knee Osteoarthritis

**DOI:** 10.1155/2012/764621

**Published:** 2012-12-27

**Authors:** Martin H. Gregory, Nicholas Capito, Keiichi Kuroki, Aaron M. Stoker, James L. Cook, Seth L. Sherman

**Affiliations:** ^1^School of Medicine, University of Missouri, Columbia, MO, USA; ^2^Missouri Orthopaedic Institute, 1100 Virginia Avenue, DC 953.00, Columbia, MO 65212, USA; ^3^Comparative Orthopaedic Laboratory, College of Veterinary Medicine, University of Missouri, Columbia, MO, USA

## Abstract

Knee osteoarthritis remains a tremendous public health concern, both in terms of health-related quality of life and financial burden of disease. Translational research is a critical step towards understanding and mitigating the long-term effects of this disease process. Animal models provide practical and clinically relevant ways to study both the natural history and response to treatment of knee osteoarthritis. Many factors including size, cost, and method of inducing osteoarthritis are important considerations for choosing an appropriate animal model. Smaller animals are useful because of their ease of use and cost, while larger animals are advantageous because of their anatomical similarity to humans. This evidence-based review will compare and contrast several different animal models for knee osteoarthritis. Our goal is to inform the clinician about current research models, in order to facilitate the transfer of knowledge from the “bench” to the “bedside.”

## 1. Introduction

Knee osteoarthritis (OA) affects an estimated 27 million Americans [1]. Despite extensive research seeking therapeutic interventions for this disease, there are still no proven disease-modifying treatments for osteoarthritis. With the number of total knee arthroplasties growing each year, this is a rapidly expanding public health epidemic, both in terms of health-related quality of life and financial expenditure [2]. The major hurdles in osteoarthritis research include elucidating the mechanisms of disease, determining methods for early detection, and developing strategies for intervention and disease modification. Translational research is a critical step towards understanding and mitigating the long-term effects of this disease process. Animal models provide practical and clinically relevant ways to study both the natural history and response to treatment of knee osteoarthritis. A translational animal model is one that facilitates the translation of findings from basic science to practical applications that enhance human health and well-being. The other types of animal models would include veterinary clinical (an animal model of an animal disease), comparative, discovery, and mechanistic, among others. This evidence-based review will compare and contrast several different animal models for knee (stifle) osteoarthritis. Our goal is to provide an outline of the factors that are important in choosing an appropriate animal model and to provide illustrative examples that demonstrate how each animal model has aided our understanding of OA. The OARSI histopathology initiative brought together experts in OA research to develop a scoring system to facilitate comparison of results across species [74–81]. The reports reviewed anatomy and histology of each animal model. We relied on these excellent reports, as well as several other review articles [82–84], to identify relevant animal models and identify areas that our review could improve upon existing work. Separate PubMed literature searches were conducted using the terms “osteoarthritis OR osteoarthrosis” and “knee OR femur OR tibia” along with “mouse,” “rat,” “rabbit,” “guinea pig,” “dog,” “sheep,” “goat,” and “horse.” Only in vivo models studie were considered. Articles were selected after a review by our multidisciplinary team of orthopedic surgeons, veterinary surgeons, and Ph.Ds. Selected articles had particular relevance to human osteoarthritis, novel findings, and ability to serve as illustrative examples of the strengths and weaknesses of a specific animal model. Admittedly, plenty of excellent articles were not included in our review. Our goal, however, was not to provide an exhaustive list of every work published on translational OA research. Our purpose was to highlight the best examples in the literature to aid the clinician/scientist in choosing an animal model for a specific research question. Our goal is to inform the clinician about current research models, in order to facilitate the transfer of knowledge from the “bench” to the “bedside.”

## 2. Factors in Determining an Appropriate Animal Model

 Unfortunately, there is no single gold standard animal model for knee osteoarthritis. Each animal model has unique advantages and disadvantages (Table 1). As such, it is critically important to understand the clinically relevant question under investigation and the hypothesis being tested, in order to choose an appropriate model. For example, if one wanted to study the natural history of knee osteoarthritis, a rat model would be inappropriate since this does not typically occur in wild-type rats [85]. In contrast, dogs or horses would be appropriate selections for this experimental design, as these animals share similar risk of knee osteoarthritis secondary to trauma, meniscal tears, osteochondrosis, and aging as their human counterparts [86, 87].

The method of osteoarthritis induction is another important consideration. While surgical anterior (cranial) cruciate ligament (ACL) transection has been the most commonly used animal model for osteoarthritis, particularly in dogs, it may not be the most applicable to human OA. In comparison to human knee OA, ACL transection is associated with immediate and severe joint instability that results in extended periods of complete lack of weightbearing on the affected limb [47, 48, 58]. In contrast, human knee OA tends to develop more insidiously, sometimes without evidence of prior injury [88, 89]. Another major criticism with respect to applicability of the ACL transection model involves the high degree of variability noted in outcome measures typically employed [49–51, 90–92]. In dogs, level of function, diagnostic imaging findings, presence and severity of meniscal pathology, and arthroscopic, gross, and histologic measures of articular cartilage damage could be variable among animals after ACL transection [49–51, 92, 93]. This results in a requirement for higher numbers of animals to be included in order to adequately power the study and appropriately apply the data to the human clinical situation. As such, costs increase greatly and use of research animals is not minimized as desired. Therefore, models of OA that more closely mimic human knee OA have been developed and tested [48, 57–60, 94]. Surgical creation of articular surface lesions or meniscal deficiency of various types as described below currently provide the most consistent and least variable models of human knee OA [48, 57–60, 94].

 Age, size, and gender/reproductive statuses of the animals also have important influences on study design and data application [95–97]. It is generally preferable to use skeletally mature animals for OA models in order to minimize the effects of intrinsic healing and regenerative capacity such that data are more applicable to humans [84]. In general, small animals (mice, rats, rabbits, and guinea pigs) are most advantageous in terms of costs, housing, genetic manipulation, and public perception, whereas large animals (dogs, goats, sheep, pigs, and horses) are most advantageous in terms of anatomical and biomechanical similarities to humans, ability to use routine diagnostic imaging, capabilities for arthroscopic interventions, and postoperative management with respect to OA research. As such, small animal models of OA are primarily used for investigating specific disease mechanisms or initial screening of therapeutics while large animal models provide more clinically relevant data and are typically required for FDA approval of diagnostics, biologics, and devices. Gender/reproductive status may also play roles in OA research in terms of hormonal influences on physiology, response to treatments, and various side effects [95]. All of these factors have direct impact on financial costs of OA research. Animal-related costs encompass not only the acquisition of research animals, but also housing of animals and salaries of personnel involved in animal care and assessments.

 Noninvasive outcome measures of disease progression are particularly important in OA research because humans remain asymptomatic until relatively late in the disease process. Many treatments that have shown promise in animal models produce disappointing results in humans because treatments are started so late in the disease course [98, 99]. Matrix metalloproteinase inhibitors reduced cartilage degradation in rats [16], guinea pigs [23], and dogs [100]. In a human clinical trial, however, a matrix metalloproteinase inhibitor failed to reduce joint space narrowing and led to significant musculoskeletal adverse effects [98]. Similarly, bisphosphonates reduce cartilage degradation in rats [12] and rabbits [21], but failed to reduce symptoms or joint space narrowing in humans [99]. There is hope that earlier detection of knee OA may allow for treatments designed to halt or even reverse disease progression. Biomarkers and magnetic resonance imaging are two areas of active research that will be discussed in detail in later sections. MRI allows noninvasive assessment of objective outcomes such as cartilage volume that can also be measured in humans [101–103]. In humans, MRI can assess cartilage volume, bone marrow lesions, ligamentous and meniscal pathology, and synovial fluid volume and synovitis [101, 102]. Several recent clinical trials used parameters assessed by MRI as outcome variables [104–106]. Dogs, goats, and horses can be imaged effectively using 1.5 and 3T clinical magnets with routine coils and sequencing [92, 93, 103, 107, 108]. Mice, rabbits, and guinea pigs are too small to undergo routine MRI in vivo [18, 24, 25, 109], and only recently has the feasibility of micro-MRI been demonstrated in rats [110].

 Finally, the public has become increasingly concerned about the humane treatment of animals. Dogs in particular have a special connection to humans and their use in medical research has drawn the scrutiny of many people. The Animal Welfare and Horse Protection Acts was an essential step in assuring that research animals are treated humanely. The study must be carefully designed to use as few animals as possible to answer a particular research question. This involves undertaking power analyses prior to using an animal model. In addition, investigators are committed to searching for ways to minimize the use of animals by adopting alternative methods when available. All animal research must be described in an Animal Care and Use Committee (ACUC) Protocol Form, which must be approved by the institutional ACUC prior any animal work being performed.

## 3. Mouse

Due to its ease of use and low cost, mouse models have served as the foundation of biomedical animal models for a long time [111]. In osteoarthritis research, however, where load and biomechanics are deemed important contributing factors, the mouse knee is problematic because of its extremely small size compared to humans [77]. Mouse knee cartilage is very thin, lacking discernible radial, transitional, and superficial layers [77, 112]. Humans and other large animals have a thin layer of calcified cartilage adjacent to the subchondral bone, whereas in mice this layer makes up a greater share of the cartilage thickness [77]. This thin cartilage makes it difficult to induce small defects that progress slowly to OA [77]. One advantage of the small joint is that slides can be made that capture an entire slice of the joint. In addition to transgenic models described below, meniscal destabilization is used to induce osteoarthritis [113]. Postoperative management is difficult with such a small animal. Functional assessments, exercise regimens, and splinting are difficult or impossible to do. Mice are too small to undergo routine MRI, but micro-MRI can be used [109].

Despite these limitations, mouse models have proved useful in elucidating the genetic and molecular pathogenesis of OA. The entire mouse genome has been sequenced and knockout mice have been used extensively to study genotype-phenotype relationships in OA. Since most cases of OA are likely to be caused by many different alleles each conferring a small amount of risk, mouse models were initially useful in studying rare monogenic disorders with OA as part of the phenotype [3–5]. For example, Kniest and Sickler syndromes were found to be associated with defects in collagen type II gene (*COLA21*) [3]. Mouse models showed that internal *COLA21* deletion led to a range of phenotypes that encompass Kniest and Sickler syndromes [6]. Mice with milder phenotypes developed significant OA as they got older. These and other studies helped to illuminate the essential role of structural proteins in skeletal development and degenerative diseases such as OA.

 More recently, mouse models have provided a means to study candidate genes found in large genomic association studies in humans. Several studies have found an association between a single-nucleotide polymorphism in the 5′-UTR of the growth and differentiation factor 5 (GDF5) gene and risk of OA [114–116]. Daans et al. used a transgenic mouse with a mutated *GDF5* allele (*Gdf*5^*Bp*−*J*/+^) to study susceptibility to OA [7]. They evaluated the development of OA using 4 models: (1) injected collagenase; (2) injected papain; (3) meniscal destabilization; and (4) exercise induced. In the collagenase model, the contralateral limbs of *Gdf*5^*Bp*−*J*/+^ mice had more OA-like changes compared to wild type (WT). The authors hypothesized that the collagenase injection led to increased loading in the contralateral limb and that *Gdf*5^*Bp*−*J*/+^ mice were more susceptible to damage from this increased load than WT mice. The running model was associated with increased synovial hypertrophy, but the papain and meniscal models did not show any difference in degenerative changes [7].

## 4. Rat

Rats offer similar advantages and disadvantages to the mouse. They are inexpensive and easy to care for. Rats have an advantage over mice in that their cartilage is thick enough to induce both partial and full-thickness cartilage defects [76, 117, 118]. This has allowed the rat to be used for the study of cartilage restoration techniques such as gene therapy [117], stem cell transplantation [118], and growth factor treatments [119]. Although larger than mice, rat joints are still extremely small compared to larger animals and humans. In contrast to mice, genetically engineered rats are not currently available. As in mice, postoperative management is difficult. Rats cannot undergo routine MRI, but recently the feasibility of micro-MRI has been demonstrated [110]. Methods to induce OA in rats include medial meniscus tear [8–10], ACL tear [12–14], partial medial meniscectomy [11], ACL tear with partial medial meniscus tear [13, 15], and iodoacetate injection [16, 17].

Rat models have been used to extensively characterize OA-related pain and evaluate treatments. Bove et al. transected the medial meniscus and MCL to characterize the pain profile of OA in rats [9]. Rats with induced OA had increased joint pain and tactile allodynia. The authors argued that this mimicked the nociceptive and neuropathic aspects of OA-related pain in humans, and thus the rat could be used as a model to study pain treatments. Fernihough et al. compared the pain profile in a partial medial meniscectomy model and iodoacetate model [10]. Both produced significant mechanical hyperalgesia and tactile allodynia, although the iodoacetate model produced more pain. The iodoacetate model has been used most often to study pain in rat models. Novel pain therapies have been tested using rat models of OA, including a histamine H3 receptor antagonist [120], an endocannabinoid hydrolysis inhibitor [26], and proteasome inhibitor [121].

## 5. Rabbit

The rabbit knee is similar in gross appearance to the human knee except for a smaller patella relative to other structures [79]. The rabbit knee, however, is kept in a higher degree of flexion and the gait is vastly different from humans and other animals [79]. Thus, the biomechanics are very different. Histologically, the rabbit is different also. The rabbit meniscus has greater cellularity than human meniscus as well as less vascular penetration [122]. Rabbits lack a lamellar collagen layer at the articulating surfaces that is present in humans and sheep [122]. A major problem in using the rabbit model is rabbit articular cartilage that is capable of regeneration, which may be due to the fact that rabbits up to 8 months of age can have open physes on growth plates in the distal femur and proximal tibia [123, 124]. Rabbit models of OA include ACL tear, [18, 19] meniscectomy [20] and chemically induced [21, 22]. Similar to mice and rats, postoperative management is difficult. Micro-MRI is required for rabbit imaging, although some lesions can be seen with routine MRI [18, 125].

Despite its limitations, the rabbit model has been used to evaluate the efficacy of various compounds. It has allowed mechanisms of efficacy to be studied in ways that would be impossible in humans. Various types of hyaluronic acid have been tested. High molecular weight hyaluronic acid was shown to be superior to lower MW [126], and cross-linked hyaluronic acid was superior to other compositions [127]. It was shown to reduce apoptosis and nitric oxide production, with the results being more pronounced earlier in the disease process [19]. Hyaluronic acid reduced expression of IL-1*β* and matrix metalloproteinase-3 (MMP-3) in the synovium but not in cartilage [128]. Zhang et al. recently demonstrated the efficacy of intra-articular injections of the antitumor necrosis factor antibody infliximab in a rabbit model where the ACL and medial menisci were transected [129]. Other compounds including caspase inhibitors [130], chitinous materials [131, 132], resveratrol [133], and bisphosphonates [134, 135] have also been studied.

## 6. Guinea Pig

The guinea pig knee is similar to the human knee, but much smaller. Bone growth stops by 4 months of age, but growth plate fusion occurs several months later [78]. The guinea pig has varus alignment of the stifles (knees), which puts increased load on medial compartment [78]. It follows that the medial compartment in guinea pigs develops OA first [27, 136]. The central portion of medial compartment is not covered by meniscus, making it more susceptible to OA [136].

**Figure 1:**
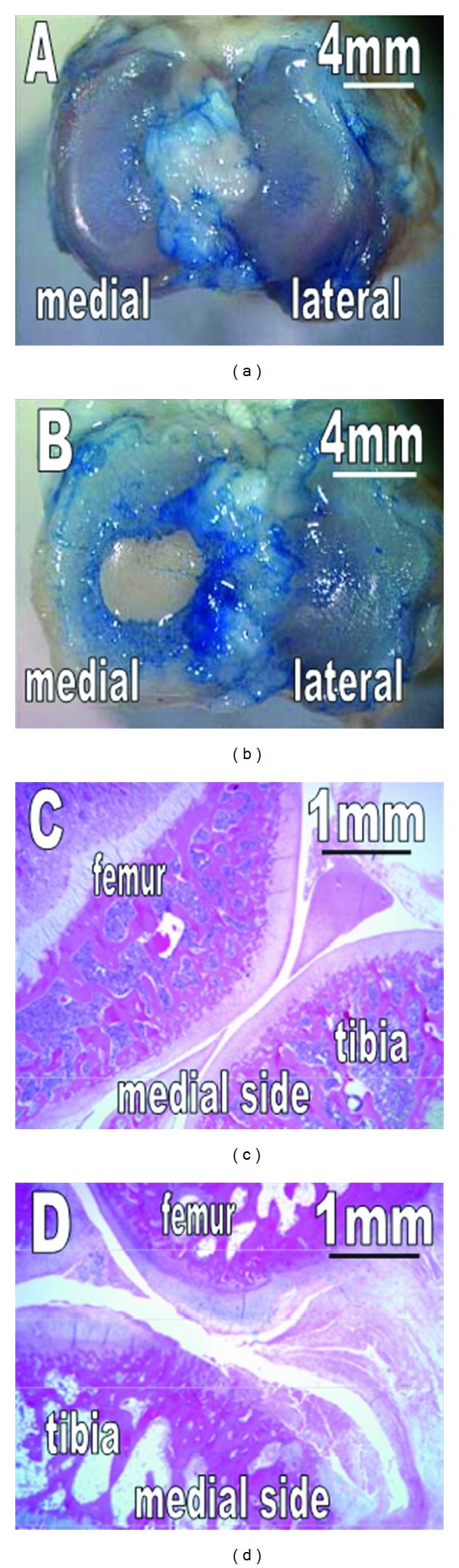
Macroscopic observation and histology of the tibial cartilage of a 3-month-old (a, c) and 12-month-old (b, d) Dunkin Hartley guinea pigs. No cartilage degeneration is observable at 3 months. In contrast, at 12 months, the cartilage is degenerated with erosion and fragmentation on the medial side. Reprinted with permission from [25].

There are two major advantages of the guinea pig as a translational model. First, the histopathology is remarkably similar to human OA [78, 137]. Second, spontaneous OA-prone animals are available (Figure 1). The Dunkin-Hartley strain [27] is the most commonly used, but there are other lesser known strains such as strain 13 [28, 29] and GOHI models [30]. These strains develop OA slower than the Dunkin-Hartley model and thus provide a useful comparison for temporal patterns of OA development [29, 30]. The much shorter time to skeletal maturity is an advantage over other spontaneous OA models such as the dog and horse. In addition, the guinea pig's docile nature makes it easy to use. A disadvantage is that while the sedentary lifestyle of the guinea pig makes it easier to care for, it is not ideal for studying the role of exercise in OA.

Much of the OA research has utilized the Hartley strain, although other models have been used, namely, medial meniscectomy [43] and chemically induced by iodoacetate [44] and papain [45]. The spontaneous OA model has allowed characterization of the natural history of OA grossly, histologically, and biochemically. Type II collagen, normally found in attachment sites of tendons and ligaments, is deposited in central portion of the posterior cruciate ligament prior to development of OA [31]. The medial meniscus ossifies in the development of OA and correlates with the severity of lesions [32, 33]. Wei et al. found an increase in proteoglycan (PG) and collagen prior to OA being seen, then a reduction in PG and collagen content with an increase in water content after OA was established [34]. This suggests articular cartilage is able to respond to increased stress up to a certain threshold and then breaks down leading to OA. Alterations in proteoglycan-collagen interactions precede changes in the articular surface [35]. MRI has been used in guinea pigs to characterize the progression of spontaneous OA [24, 25, 36–38].

Advanced age and body mass index (BMI) are risk factors for OA in humans, and guinea pig models have allowed these risk factors to be studied more in depth. Bendele and Hulman demonstrated the importance of BMI by showing that diet reduction reduced the severity of OA lesions [96]. Hyttinen et al. found that alterations in collagen structure in response to exercise differed in young and old guinea pigs [97].

The spontaneous OA model is an advantage over injury-induced models because it allows study of prophylactic therapy, such as matrix metalloproteinase inhibitors [23] and glucosamine and chondroitin sulfate [39]. Additionally, the Hartley model is useful to study pain from OA, since the surgery itself may cause pain in induced models of OA. Endocannabinoid hydrolysis inhibitors have recently been tested in guinea pig models [26]. The feasibility of newer therapies such as RNA interference [40], gene therapy [41], and human mesenchymal stem cells [42] has also been evaluated in guinea pig models.

## 7. Canine

**Figure 2:**
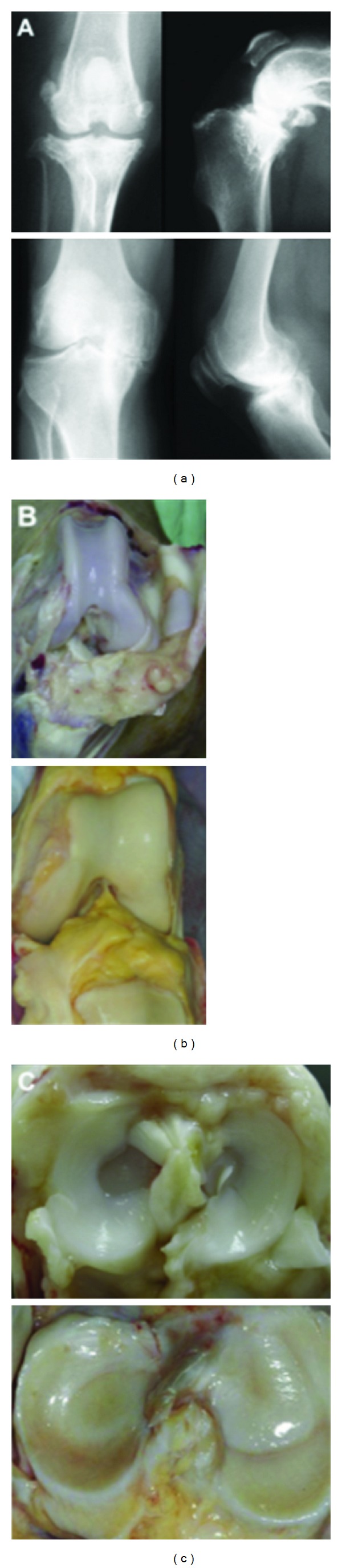
Comparative radiographic (a) and gross (b, c) anatomy of the canine stifle (top row) and human knee (bottom row). The radiographic images show osteoarthritic joints while the gross images show normal patellofemoral joints (b) and normal tibial plateaus (c). The canine stifle joint is approximately 3.5–5 cm from medial to lateral edge for medium to large breed dogs (upper left panel a) compared to approximately 7–10 cm for the human knee joint (lower left panel a) reprinted with permission from [75].

The canine model is probably the closest to a gold-standard animal model for OA currently available. The canine stifle (knee) joint is remarkably similar to the human knee (Figure 2) [75]. The only major differences are sesamoid bones in the popliteus and lateral and medial heads of the gastrocnemius muscles, and the long digital extensor tendon that crosses the intra-articular anterolateral compartment [75]. Dogs are large enough to undergo arthroscopy and MRI. MRI can detect early signs of OA in dogs [92, 103] and is more sensitive than radiography at tracking progression of OA after ACL transection [92, 93, 103]. Dogs are by far the easiest to control postoperatively. They tolerate splinting better than other animals and are able to undergo exercise regimens, including water training. Furthermore, since canine models are commonly used, there are a wealth of data and many validated outcome measures that allow comparison across studies. Examples are the Canine Brief Pain Inventory [138] and force plate gait analysis [139]. Like the mouse, the canine genome has been sequenced [140].

The major disadvantages of the canine model relate to cost and public perception. Dogs are relatively expensive particularly if bred for research purposes. Housing requirements are more costly compared to smaller animals, including daily walking requirements. Humans have a special connection to dogs, and this has stirred strong opposition to the use of dogs in research.

The first canine model was developed several decades ago by Pond and Nuki [47]. They transected the ACL in dogs and found radiographic and histologic features of OA. Since then the Pond-Nuki model has been used extensively [48–55, 59, 100]. Other canine models include meniscectomy or meniscal release [48, 57], creation of a focal defect [48, 58–60], or chemical induction [141, 142]. In addition, dogs develop spontaneous osteoarthritis [86]. Osteoarthritis is common for veterinarians to see, with an estimated 20% of dogs older than one year of age affected [143]. Dogs undergo similar treatments as humans, such as oral therapy, intra-articular hyaluronic acid injections, arthroscopic surgery, and knee replacement [144, 145]. As with the guinea pig, this is a tremendous advantage over other animals because it enables study of the natural progression of the disease.

Having multiple models at the researcher's tool kit allows examination of subtle differences in the pathology associated with different causes of OA. Kuroki et al. examined the subchondral bone changes in ACL transection, medial femoral condylar groove creation, and meniscal release compared to sham surgery [48]. ACL transection led to considerably more cartilage damage, functional loss, and thinnest trabecular bone. Liu et al. found that proteoglycan levels differed in ACL and spontaneous OA models [52]. Notably, aggrecan content decreased in spontaneous OA, but increased after ACL transection. Marijnissen et al. found that a groove model led to greater cartilage damage but less synovial inflammation compared to an ACL model [59]. These results show that OA progression can be varied by the type of insults and the method of inducing OA needs to be carefully considered and generalization of results to humans should be done cautiously.

Another advantage of canine models is their similar gastrointestinal physiology to humans, which facilitates the study of enteral therapies. Licofelone, a novel drug that inhibits cyclooxygenase and 5-lipoxygenase, illustrates the use of dogs as a translational model. Its efficacy was demonstrated by reducing the size of cartilage lesions after ACL transection in dogs [53]. Using a canine model allowed more detailed analysis of the effects on the knee joint, including its effects on cartilage, subchondral bone, and the synovium. It reduced expression of matrix metalloproteinase 13 (MMP-13), cathepsin K, and aggrecanases in cartilage [54]. It attenuated subchondral bone loss, which also showed reduced expression of MMP-13 and cathepsin K [55]. It reduced the size of villous hyperplasia in the synovium and synthesis of collagenase 1 and IL-1*β* [53].

One of the concerns about the “translatability” of animal models is that the benefit of treatments may be greater in animals because treatment is initiated at the time the injury is induced, before the development of OA. Humans are treated much later on in the disease process. While the ideal way to address this would probably be to take advantage of the canine's tendency to develop spontaneous OA, Moreau et al. attempted to address it by delaying licofelone until 4 weeks after ACL transection, showing that licofelone was still effective [56]. Licofelone has recently been studied in humans and has shown potential as a disease-modifying agent [104].

**Figure 3:**
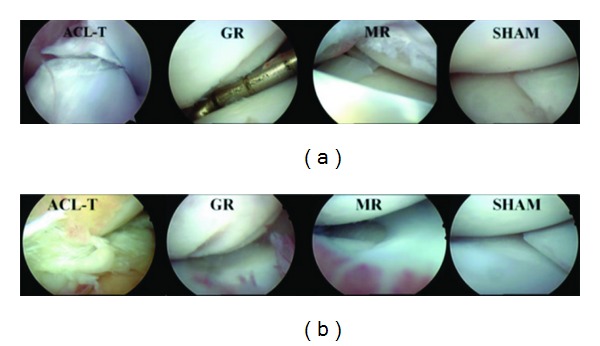
(a, b) Initial and 12-week postoperative arthroscopic views of one dog from each surgical induction model. ACL-T, anterior cruciate ligament transection; GR, groove model; MR, meniscal release; SHAM, manipulation without insult reprinted with permission from [46].

A recent study by Garner et al. illustrates the advantages of the canine as a translational model [46]. Previous studies with cartilage explants found increased metalloproteinase expression in OA patients compared to normal patients [146, 147]. Another study found differential chemokine expression in OA patients [148]. In particular, monocyte chemoattractant protein 1 (MCP1) was elevated compared to normal. Garner et al. simultaneously examined 3 different induction methods of OA as well as spontaneous OA to identify potential biomarkers of OA [46]. They used arthroscopy to induce OA by ACL transection, meniscal release, and groove creation, with another group of dogs undergoing a sham operation (Figure 3). The second part of the study examined dogs presenting to a veterinarian for surgical treatment of OA. A group of dogs without evidence of OA was used as a control. They tracked a variety of potential markers, including various matrix metalloproteinases and cytokines. They found that MCP-1 and IL-8 were elevated in the synovial fluid in all of the induced OA models and spontaneous OA. Each exhibited high sensitivity and specificity for detecting OA [46]. The results need to be applied to humans, but the success of the biomarkers in differentiating OA from normal in induced OA and spontaneous OA is a promising development in the search for OA biomarkers.

## 8. Goats and Sheep

There are several characteristics of goats and sheep that facilitate their use as translational models. Their knee joints are closer in size to humans compared to dogs and smaller animals [80]. Thus, arthroscopy and MRI are feasible [107, 149]. Goats and sheep are fairly easy to use, as they are generally not aggressive. The gross anatomy of the joint is similar to humans, except a long digital extensor tendon crosses within the joint in the anterolateral compartment [80].

 One disadvantage in using goats and sheep for OA models is that they are not prone to spontaneous arthritis. Surgical partial or complete meniscectomy is generally used to induce OA, as ACL transection causes only limited cartilage damage in the goat [150]. This is in contrast to canines, where ACL transection causes significantly more damage than meniscal release or groove creation [48]. Another disadvantage is that goats and sheep are ruminant rather than monogastric, which is not ideal for studying enteral therapies.

Sheep and goat models have been useful in exploring the effects of meniscal pathology in relation to OA. Bylski-Austrow et al. showed that meniscectomy leads to a significant increase in joint pressure, but that the joint pressure decreased over time [61]. Others have shown that exercise exacerbates osteoarthritic changes in sheep without menisci [62, 63]. Beveridge et al. conducted a detailed analysis of the kinematics of the sheep stifle (knee) following lateral meniscectomy [64]. The minimum tibiofemoral distance shifted laterally, leading to more cartilage damage in that area. More recently, techniques for replacing the meniscus have been explored using ovine models. Kelly et al. demonstrated the chondroprotective effects of meniscal allograft transplantation in a sheep model [65]. Murphy et al. found that intra-articular injection of mesenchymal stem cells suspended in hyaluronan led to some regeneration of excised meniscus and slowing of OA progression [66].

Ovine models have also been used to study novel therapies to repair cartilage defects. Heiligenstein et al. demonstrated that genetically modified chondrocytes implanted into ovine cartilage defects expressed genes for 21 days [151]. Marquass et al. successfully implanted predifferentiated mesenchymal stem cells that remained free of degradation after one year [152]. They compared these results to implantation of chondrocytes and found favorable results. A novel technique for autologous chondrocyte transplantation in a single procedure showed success up to 6 months in a goat model [153].

## 9. Horse

The front “knee” of the horse is actually analogous to the human wrist with two layers of carpal bones. The metacarpophalangeal joint, involving the large canon bone (third metacarpal) and the first phalanx, is most susceptible to spontaneous osteoarthritis [81]. The most commonly used osteochondral fragment-exercise model involves creating fragments in the middle carpal joint [68]. An advantage of this model is that no instability is created, but the cartilage is thinner compared to human knee cartilage [81].

Horses are unique in that they are not solely “translational” models, having long been known to suffer from spontaneous OA [87]. The lucrative horse racing industry has made finding effective treatments for joint pathology extremely valuable. It follows that much of the equine research has been devoted to that applicable to athletics. Bolam et al. demonstrated that a single traumatic event can lead to osteoarthritis by arthroscopically inducing impact injuries to the articular surface of the medial femoral condyle [69]. Studies have indicated that short-term immobilization has minimal effects on joint health [154], but 7 weeks of immobilization led to reduced range of motion, reduced bone mineral density, and increased lameness even after 8 weeks of increasing exercise [155, 156]. Frisbie et al. found that several synovial fluid biomarkers and six serum biomarkers were elevated in an osteochondral fragment-exercise model compared to exercise alone [70]. Auer et al. examined the efficacy of hyaluronic acid using experimentally induced OA and spontaneous OA [67]. Other therapies such as triamcinolone acetonide [71, 72] and betamethasone [68] have been also tested in equine models. Recently, new technologies have been evaluated in horses. Frisbie et al. demonstrated the feasibility and efficacy of using an adenoviral vector to express interleukin 1 antagonist in the joints of horses with osteoarthritis [73]. The same lab demonstrated the repair of cartilage defects using chondrocyte implantation using only a single procedure [157].

## 10. Conclusion

Knee osteoarthritis remains a tremendous public health concern, both in terms of health-related quality of life and financial burden of disease. Translational animal research plays a critical role in helping to understand the mechanism of disease, to improve methods of early detection, and to identify and investigate potential treatment targets. The choice of a particular animal model is multifactorial. Smaller animals are easier to use and less costly, but the information gathered may be less applicable to human OA. Larger animals offer the advantages of spontaneous or readily inducible OA, non or minimally invasive evaluation techniques (i.e., arthroscopy, MRI, and biomarkers) without the need for immediate sacrifice, and closer similarity to human knees. Disadvantages include cost and public perception. Each model has contributed to our understanding of OA (Table 2). Future translational animal models will focus on complete elucidation of disease pathogenesis, determining early markers of disease, and ultimately developing disease-modifying therapy for knee OA.

## Figures and Tables

**Table 1:** Advantages and disadvantages of osteoarthritis animal models.

	Advantages	Disadvantages	Models used
Mouse	Low cost Easy of use Genome sequenced Can view hole knee can on slides	Thin cartilage Postoperative management difficult	Genetic [3–7] Meniscal destabilization [7] Chemical [7]

Rat	Low cost Ease to use Thicker cartilage than mouse Can view hole knee can on slides	Small joints Postoperative management difficult	Medial meniscus tear [8–10] Partial medial meniscectomy [11] ACL transection [12–14] ACL transection with partial medial meniscectomy [13, 15] Chemical [16, 17]

Rabbit	Easy to use	Knee biomechanics Cartilage capable of regeneration Different histology from human Postoperative management difficult	ACL transection [18, 19] Meniscectomy [20] Chemical [21, 22]

Guinea pig	Similar histopathology to human Prone to spontaneous OA	Sedentary lifestyle Arthroscopy not possible	Spontaneous [23–42] Meniscectomy [43] Chemical [44, 45]

Dog	Prone to spontaneous OA Arthroscopy feasible MRI feasible GI physiology Genome sequenced Validated outcome measures	Cost Public perception	Spontaneous [46] ACL transection [46–56] Meniscal release [48, 57] Focal cartilage defect [48, 58–60]

Sheep/goat	Large joint Easy to use Arthroscopy feasible MRI feasible	Cost GI physiology	Partial/total meniscectomy [61–66]

Horse	Spontaneous OA Can induce OA without instability Arthroscopy feasible MRI feasible	Cost Anatomy	Spontaneous [67] Osteochondral-fragment exercise model [68–73]

**Table 2:** Summary of contributions to osteoarthritis knowledge.

Mouse	Mutations in structural proteins can lead to OA [6]. Single-nucleotide polymorphism associated with increased risk in human populations leads to increased OA in a mouse model [7].

Rat	Novel therapies shown to reduce pain [26, 120, 121]. Feasibility of new cartilage restoration techniques demonstrated [117–119].

Rabbit	Hyaluronic acid (HA) more effective earlier in disease process [19]. HA reduces inflammatory cytokine and metalloproteinase expression in synovium but not cartilage [128]. High molecular weight hyaluronic acid superior to low MW [126]. Infliximab may be efficacious in OA [129].

Guinea pig	Structural alterations occur in the meniscus and posterior cruciate ligament (PCL) prior to development of OA [31–33]. Collagen and proteoglycan content increases prior to development of OA and then decreases once OA is evident [34]. Diet reduction reduces severity of OA lesions [96].

Dog	OA progression differs based on inciting event [48, 52, 59]. ACL transection leads to severe damage compared to meniscal release and groove creation [48]. Oral therapy reduces metalloproteinase expression in the joint and reduces cartilage lesions [53–55]. Biomarkers in synovial fluid had high sensitivity and specificity in diagnosing OA [46].

Sheep/goat	Knee joint remodels to reduce acute increase in joint pressure from meniscectomy [61]. Exercise exacerbates OA changes after meniscectomy [62, 63]. Meniscal allograft reduces cartilage damage [65].

Horse	Single traumatic event can lead to OA [69]. Short-term immobilization has minimal effects on joint health, but long-term immobilization reduces bone mineral density [154–156]. Chondrocyte implantation feasible in single surgery [157].
